# Drop-Dry Deposition of SnO_2_ Using a Complexing Agent and Fabrication of Heterojunctions with Co_3_O_4_

**DOI:** 10.3390/ma16155273

**Published:** 2023-07-27

**Authors:** Tong Li, Masaya Ichimura

**Affiliations:** Department of Electrical and Mechanical Engineering, Nagoya Institute of Technology, Gokiso, Showa, Nagoya 466-8555, Japan; cmc13210@nitech.jp

**Keywords:** drop-dry deposition, SnO_2_, Co_3_O_4_, heterojunction

## Abstract

The drop-dry deposition (DDD) is a simple chemical technique of thin film deposition, which can be applied to metal oxides. The deposition solution is an aqueous solution including a metal salt and an alkali. However, some metal ions react spontaneously with water and precipitate. This work is the first attempt to use complexing agents in DDD to suppress the precipitation. SnO_2_ thin films are fabricated using DDD with Na_2_S_2_O_3_ as a complexing agent and via annealing in air. The results of the Auger electron spectroscopy measurement show that the O/Sn composition ratio of the annealed films approached two, indicating that the annealed films are SnO_2_. The photoelectrochemical measurement results show that the annealed films are n-type. Co_3_O_4_/SnO_2_ heterojunction is fabricated using p-type Co_3_O_4_ films which are also deposited via DDD. The heterojunction has rectification and photovoltaic properties. Thus, for the first time, a metal oxide thin film was successfully prepared via DDD using a complexing agent, and oxide thin film solar cells are successfully prepared using only DDD.

## 1. Introduction

Solar energy is an inexhaustible renewable energy source. Photovoltaic technology that uses solar energy is an environment-friendly and promising technology. In addition to crystalline silicon solar cells, thin-film materials such as CdTe [1], GaAs [2], and Cu(In,Ga)Se_2_ (CIGS) [3] have been widely studied. In this study, we focus on metal oxide materials.

Metal oxides are an important branch of inorganic materials. Their unique physical and chemical properties lead to a wide range of applications, including electronic devices, batteries, catalysts, and sensors. Many metal oxide materials consist of non-polluting and abundant elements, meeting the requirements of inexpensive production. From an optical point of view, there are many metal oxide materials suitable for photovoltaic applications. All-oxide photovoltaics cells are promising to realize extremely cheap photovoltaic systems.

This study focuses on tin oxide (SnO_2_). SnO_2_, having a rutile structure, is an n-type semiconductor with a wide bandgap (>3.6 eV) [4,5]. Due to its excellent stability, transparency and electrical conductivity, it is usually used as a transparent conductive oxide (TCO) [6,7,8,9,10]. SnO_2_ can also form p-n junctions with p-type semiconductors and is utilized in sensors, diodes, and other fields [11,12]. In the field of solar cells, Vequizo et al. fabricated SnS/SnO_2_ heterojunctions via electrochemical deposition (ECD) for photovoltaic application [13]. Qin et al. prepared Cu_2_O/SnO_2_ heterojunction solar cell via the electron beam evaporation technique [14]. SnO_2_ thin films also have many applications as dye-adsorbed films of dye-sensitized solar cells or compact layers of perovskite solar cells [15,16,17,18,19,20,21].

The methods of preparing SnO_2_ thin films include chemical bath deposition (CBD) [22,23], magnetron sputtering [11,13], spray pyrolysis [12], sol–gel technique [24,25,26], ECD [18,27,28], etc. In this work, we prepare tin hydroxide thin films using the drop-dry deposition (DDD) method [29,30,31], and transform it into SnO_2_ films via annealing. DDD is a method of preparing thin films by dropping the deposition solution on a substrate and then drying it. The apparatus is simple, requiring only a pipette and a heating plate. Physical and chemical vapor depositions mostly require vacuum or leak-tight reactors, and the equipment is expensive. Compared with these methods, the equipment required for DDD is simple and easy to operate. Compared with other chemical liquid phase deposition methods, DDD has some advantages, as described below.

So far, Mg(OH)_2_, Co_3_O_4_ and NiO have been successfully fabricated using DDD [29,30,31]. For example, we prepared a 0.5 μm thick Co(OH)_2_ thin films via DDD with a total deposition time of about 30 min [30]. The deposition time in DDD is much shorter than for CBD; the deposition time is usually several hours in CBD [32]. Spray pyrolysis is another popular chemical technique, but it requires an atomizer and high temperatures (300–450 °C) [33]. For DDD, the equipment is simpler (just a pipette), and thin films can be deposited at lower temperatures (60 °C). The experimental equipment for DDD is also simpler than those of the spin-coating: spin-coating requires a spin coater. In general, the advantages of DDD are simple equipment, easy operation, low cost, low deposition temperature, and short deposition time.

In the previous studies on DDD, the deposition solution used was an aqueous solution including a metal salt (e.g., Co(NO_3_)_2_) and an alkali (e.g., NaOH). The metal hydroxide is generated in the solution, and the solution is saturated or nearly-saturated without precipitation by controlling the concentration of the chemicals and the pH in the solution. However, the deposition solution of tin hydroxide cannot be prepared similarly. When a tin salt (e.g., SnSO_4_) is dissolved in water, hydrolysis occurs and tin hydroxide precipitates even without adding alkali [34], making the solution unusable for deposition.

To prevent precipitation in the solution, a complexing agent is added to the DDD solution for the first time in this work. If Sn^2+^ ion forms a complex, the hydrolysis reaction will be suppressed. There are many types of complexing agents, and chemicals such as EDTA and tartaric acid are commonly used in metal plating. Studies on complexes based on Na_2_S_2_O_3_ have also been reported [35]. In this work, tartaric acid and Na_2_S_2_O_3_ are used to prepare the deposition solutions.

In this work, we also fabricate Co_3_O_4_/SnO_2_ heterojunction solar cells with Co_3_O_4_ prepared via DDD. Co_3_O_4_ is a p-type metal oxide semiconductor with two direct bandgaps of 1.5 eV and 2.0 eV, and is well suited for an absorber material in solar cells [36]. Co_3_O_4_ has already been used in photovoltaic devices [37,38,39,40]. So far, Co_3_O_4_/SnO_2_ heterojunctions have been mainly used in gas sensors [41,42,43,44] and catalysts [45,46,47], whereas we attempt to apply Co_3_O_4_/SnO_2_ heterojunctions in solar cells for the first time. This paper is also the first report of solar cells fabrication using only DDD. As shown below, photovoltaic output is confirmed for the Co_3_O_4_/SnO_2_ heterojunction, which demonstrates that DDD can be actually used for solar cell fabrication.

## 2. Experimental Design

**Figure 1:**
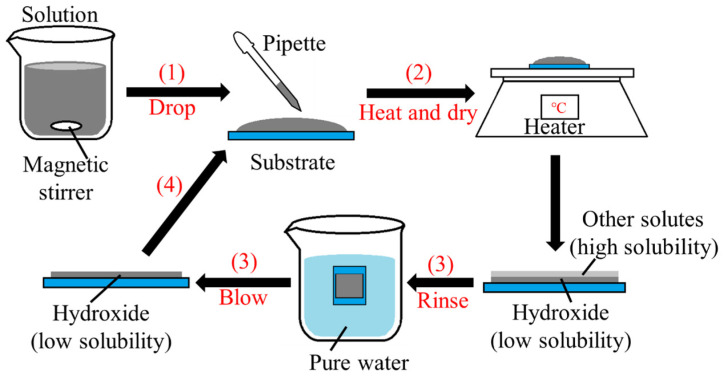
Schematic diagram of the steps for preparing thin films via DDD.

The deposition process of DDD is shown in Figure 1. In the deposition solution, tin(II) sulfate (SnSO_4_, minimum 93% purity, Kanto Chemical Co., Inc., Tokyo, Japan) was used as the Sn^2+^ source, and sodium hydroxide (NaOH, minimum 97% purity, Kanto Chemical Co., Inc., Tokyo, Japan) was added to adjust the solution pH. The SnSO_4_ concentration was 10 or 20 mM. Tartaric acid (L(+)-Tartaric acid, minimum 99% purity, Kanto Chemical Co., Inc., Tokyo, Japan) or sodium thiosulfate (Na_2_S_2_O_3_, minimum 97% purity, Kanto Chemical Co., Inc., Tokyo, Japan) was added as a complexing agent. The specific deposition solution conditions with Na_2_S_2_O_3_ addition are shown in Table 1. Fluorine-doped tin oxide (FTO)-coated glass (Furuuchi Chemical Co., Ltd., Tokyo, Japan), indium tin oxide (ITO)-coated glass (Furuuchi Chemical Co., Ltd., Tokyo, Japan) and alkali-free glass (Corning Co., Corning, NY, USA) sheets were selected as the substrates, and the deposition area was 1.8 × 1.8 cm^2^. Briefly, 0.1 mL of the solution was dropped each time, the heating temperature was 60 °C, and the deposition cycles were 5 times. After depositing the thin film, the sample was placed in a tube furnace and annealed at 200 or 400 °C for 1 h in air to convert the as-deposited film into a SnO_2_ film. For resistivity measurements, inter-digit-pattern indium electrodes (0.1 × 0.1 cm^2^) were fabricated via vacuum evaporation on the films deposited on the alkali-free glass substrate.

**Figure 2:**
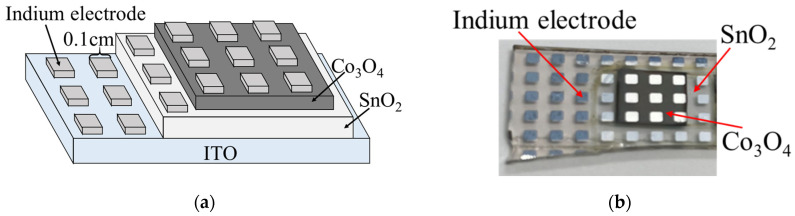
Co_3_O_4_/SnO_2_ heterojunction: (**a**) schematic diagram and (**b**) photograph.

Figure 2 shows the schematic and photograph of the Co_3_O_4_/SnO_2_ heterojunction. The Co_3_O_4_/SnO_2_ heterojunctions were fabricated as follows: First, the SnO_2_ film with an area of 1.8 × 1.8 cm^2^ was fabricated on the ITO substrate via DDD and annealing (at 200 °C, as described below). Then, a Co_3_O_4_ film was fabricated on it via DDD and annealing at 200 °C; the preparation of Co_3_O_4_ is the same as reported in ref. [30]. The deposition area of Co_3_O_4_ was limited to 0.8 × 0.8 cm^2^, and the deposition solution contained 20 mM of Cobalt(II) nitrate hexahydrate (Co(NO_3_)_2_·6H_2_O, minimum 97% purity, Kanto Chemical Co., Inc., Tokyo, Japan) and 10 mM of NaOH. In order to avoid the Co_3_O_4_ layer from becoming too thick, the deposition cycles were 3 times instead of 5 times. For the current density–voltage (J-V) characterization, indium electrodes (0.1 × 0.1 cm^2^) were fabricated on the heterojunction via vacuum evaporation.

The optical properties, structure and morphology of the films were characterized through transmittance measurements (Jasco V-570 UV/VIS/NIR spectrometer, Tokyo, Japan), X-ray diffraction (XRD) measurements (SmartLab SE X-ray diffractometer, Tokyo, Japan), scanning electron microscope (SEM) images and Auger electron spectroscopy (AES) measurements (JEOL JAMP-9500F, Akishima, Japan). All equipment was calibrated before use. Photoelectrochemical (PEC) measurement was carried out in a three-electrode system with a Ag/AgCl reference electrode. A 100 mM sodium sulfate (Na_2_SO_4_, minimum 99% purity, Kanto Chemical Co., Inc., Tokyo, Japan) solution was used as the electrolyte. The samples were intermittently irradiated with 100 mW/cm^2^ light (ABET Technologies 10500 Sun Simulator, Milford, CT, USA) at 5 s intervals and scanned for sample potentials in the range of −1 to 0 V and 0 to 1 V at a scan rate of 5 mV/s.

## 3. Results and Discussion

### 3.1. Deposition Reactions

When 10 mM of tartaric acid was added to the solution containing 10 mM of SnSO_4_, no precipitation occurred, and the solution was completely transparent. White deposits were obtained through dropping and drying of the solution on the substrate, but all the deposits were dissolved in water during the rinsing process. Thus, tin hydroxide or oxide was not successfully formed as a thin film. This occurred because complexes with tartaric acid were stable and were not decomposed to generate hydroxide during the drying process. The substance remaining on the substrate would be the complex that is easily soluble in water, and thus was dissolved during the rinsing process.

The complex with Na_2_S_2_O_3_ is expected to be less stable than that with tartaric acid, since Na_2_S_2_O_3_ is in fact not common as a complexing agent in metal plating. Solutions with different amounts of Na_2_S_2_O_3_ and NaOH were prepared and the color and precipitation of the solutions are given in Table 1.

For SnSO_4_ concentration of 10 mM, the solution with 200 mM of Na_2_S_2_O_3_ was colorless and transparent, while the solution with 100 mM of Na_2_S_2_O_3_ was light white. There was no precipitation for these two cases. When the Na_2_S_2_O_3_ amount was further reduced to 50 mM, the color of the solution deepened and became yellowish-white. Since the solution did not exhibit precipitation, it is considered that S_2_O_3_^2−^ and Sn^2+^ successfully formed the complex, as reported in ref. [35]:

Sn^2+^ + 2S_2_O_3_^2−^ = [Sn(S_2_O_3_)_2_]^2−^ (1)

The solutions of conditions I to III were dropped and dried on the substrate. The deposit under condition III (200 mM of Na_2_S_2_O_3_) was removed during the rinsing process. In contrast, for conditions I and II (50 mM and 100 mM of Na_2_S_2_O_3_), a white film remained on the substrate after rinsing. The film thickness was about 0.2 μm. Under condition IV (SnSO_4_ amount increased to 20 mM), the solution appeared to be yellow and exhibited precipitation, and thus, could not be used for depositing a thin film.

Under conditions I–IV, the solution is acidic. NaOH was added to adjust the pH of the solution (conditions V and VI). After adding 2.5 mM of NaOH (condition V), the pH of the solution was similar to that of condition IV, and the solution was yellow, showing precipitates. The pH of the solution was 11.7 after adding 25 mM of NaOH (condition VI) and the color of the solution was initially white and it changed to dark after several minutes. The color change of the alkaline solution may be due to the disproportionation reaction of the tin hydroxide to produce Sn(metal) [48], which causes the solution to become dark.

**Figure 3:**
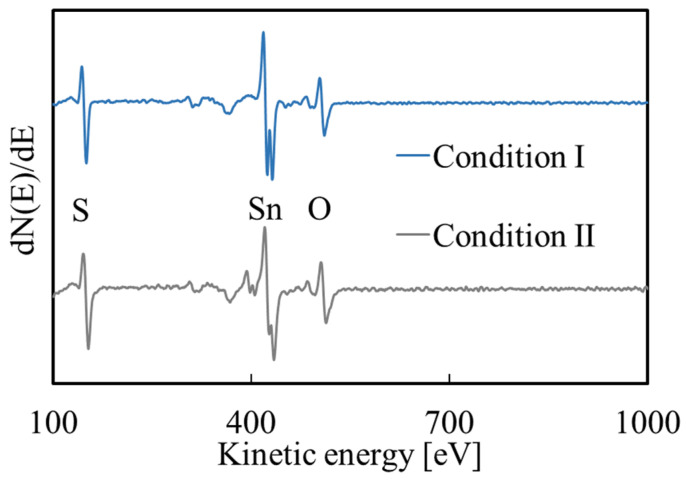
AES measurement results for the films prepared under conditions I and II.

AES measurements were performed on the films prepared under conditions I and II, and the results are shown in Figure 3. In addition to Sn and O, the films prepared under acidic environment also contain a significant amount of S. It shows that in addition to tin hydroxide, the film also contained sulfur and/or sulfide.

Based on the above results, the reactions during the deposition process could be considered as follows.

(a) Decomposition of the complex.

[Sn(S_2_O_3_)_2_]^2−^ = Sn^2+^ + 2S_2_O_3_^2−^ (2)

(b) Tin hydrolysis (formation of tin hydroxide)

(c) Tin disproportionate reaction [48].

2Sn(II) = Sn(0) + Sn(IV)(3)

(d) Decomposition of S_2_O_3_^2−^ (release of S) [49].

S_2_O_3_^2−^ + H^+^ = HSO_3_^−^ + S (4)

(e) Formation of tin sulfides.

Sn(0) + xS = SnS_x_ (5)

(f) Reaction of Sn^2+^ with OH^−^ ions (formation of tin hydroxide)

In an acidic environment, reactions (a)–(e) can occur. Because [Sn(S_2_O_3_)_2_]^2−^ is not so stable, Sn^2+^ ions will be released and react with water to generate tin hydroxide (reactions (a) and (b)). In reaction (d), S_2_O_3_^2−^ ion reacts with H_2_O or H^+^ to release S. And in reactions (c) and (e), due to the disproportionation reaction, a part of tin and S further react to form SnS_x_. The final products will be tin hydroxide, SnS_x_, and S.

In an alkaline environment, reactions (a)–(c) and (f) will occur but not reactions (d) and (e). Due to the presence of a large amount of OH^−^, Sn^2+^ ions released from [Sn(S_2_O_3_)_2_]^2−^ react with OH^−^ to generate the white precipitate of tin hydroxide (reaction (f)). And in reaction (c), metallic Sn is generated, resulting in a dark color of the solution and the precipitates. The final products will be tin hydroxide and Sn(metal).

In the characterization described below, the following deposition conditions were adopted on the basis of the above analyses: 10 mM of SnSO_4_ and 50 mM o fNa_2_S_2_O_3_ with no NaOH added (condition I).

### 3.2. SnO_2_ Film Properties

**Figure 4:**
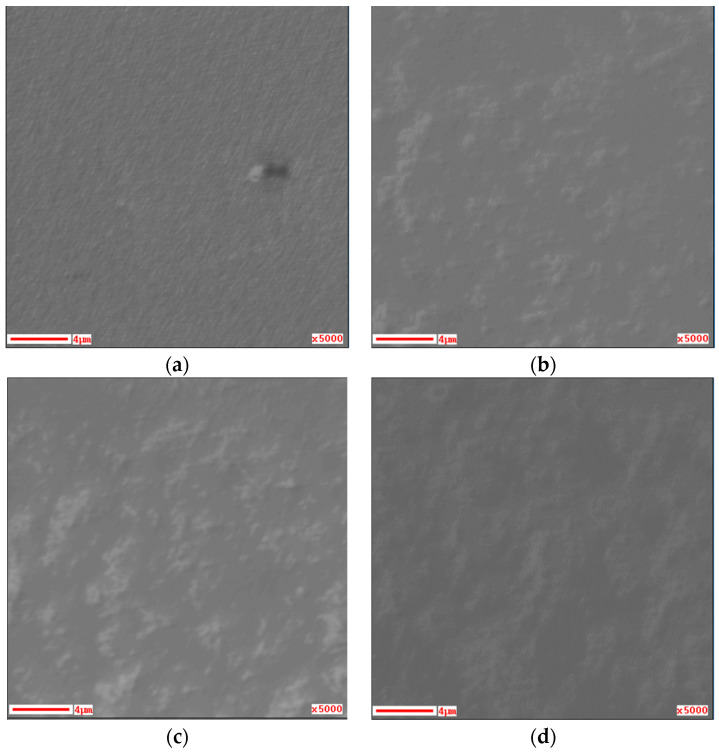
SEM images of films on the FTO substrate: (**a**) only the FTO substrate; (**b**) as-deposited; (**c**) annealed at 200 °C for 1 h; and (**d**) annealed at 400 °C for 1 h.

The film thickness was about 0.2 μm before annealing and it reduced to about 0.15 μm after annealing at 200 °C and 400 °C. This is mainly due to the conversion of hydroxides into SnO_2_. Figure 4 shows the SEM images of the films on the FTO substrate before and after annealing. Compared to the FTO substrate in Figure 4a, the film before annealing in Figure 4b clearly has more grains. In the SEM image of the SnO_2_ thin film prepared at 60 °C using CBD, it was observed that the crystal grains were spherical [22,23]. In contrast, shape of the grains in Figure 4b were irregular. The thin films prepared via DDD contain not only SnO_2_ but also substances such as SnS_x_, which may affect the formation of grains. As can be seen in Figure 4c,d, the size and number of these grains did not change significantly with the increase in annealing temperature.

Since the FTO substrate is also SnO_2_, the FTO substrate was not used for AES, XRD and PEC measurements, and instead the ITO substrate was used.

**Figure 5:**
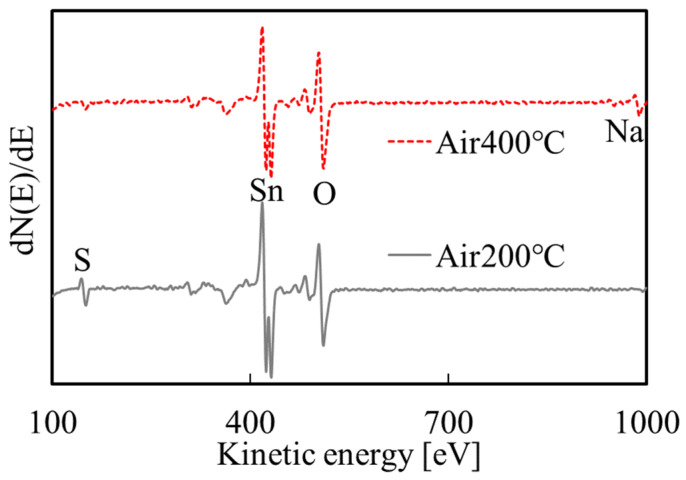
AES measurement results for the films after annealing.

Figure 5 shows the AES measurement results after annealing. The S peak intensity decreased after the annealing at 200 °C, and it further decreased after 400 °C annealing. It is presumed that this phenomenon occurs because the S in the film reacts with oxygen in the air during annealing to produce gases such as SO_2_ and then escapes. The Na signal is a result of contamination by the deposition solution. Na was not detected in the film before annealing (Figure 3) because the rinsing step removed Na from the surface of the film, while Na located deeper in the film was not removed and was exposed by the decomposition of substances such as SnS on the surface during annealing. In addition, due to the detection limit of AES (about 1%), there may also be a small amount of undetected Na in the films before annealing and after annealing at 200 °C. Taking the SnO_2_ standard sample as a reference, the O/Sn composition ratio before and after annealing was calculated: the ratio is 1.11 before annealing and it is 1.65 and 2.15 after annealing at 200 and 400 °C, respectively, as shown in Table 2. As the temperature increases, O/Sn ratio approached two, which indicates that the films annealed at 400 °C are SnO_2_.

**Figure 6:**
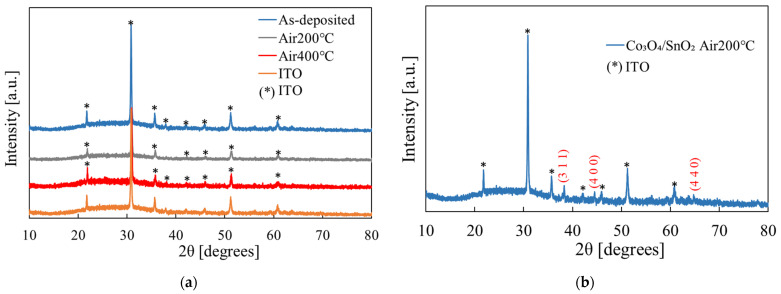
XRD results: (**a**) tin hydroxide films before and after annealing; and (**b**) Co_3_O_4_/SnO_2_ heterojunction after annealing (the indices are for Co_3_O_4_).

The structural properties of the films were characterized via XRD measurements, as shown in Figure 6. However, we did not observe any peak other than those of the ITO substrate, as shown in Figure 6a. Comparison with the powder diffraction file ICDD 00-041-1445 for SnO_2_ also shows that the peaks in Figure 6a are not from SnO_2_. Therefore, the films before and after annealing may be amorphous. The other substances mixed in the films may also affect crystallization, just as for the grain formation (SEM results).

**Figure 7:**
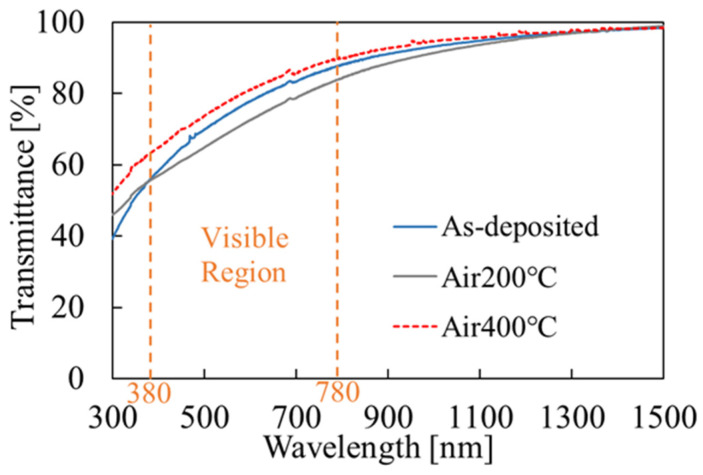
Optical transmittance measurement results for the films before and after annealing.

The results of optical transmittance measurement are shown in Figure 7. The transmittance of the film in the visible region before annealing was 55–87%, which slightly decreases to 55–83% after annealing at 200 °C, and increases to 63–89% after annealing at 400 °C. The decrease in the visible region is due to scattering by the surface roughness and absorption by the narrow-bandgap SnS_x_ phase [50,51] in the film. The literature value of the band gap is 3.6 eV for SnO_2_ [4,5] but edge around the there is no clear absorption corresponding wavelength (340 nm). This would indicate the amorphous nature of the film. The transmittance of the films annealed at 400 °C is higher than that of the films annealed at 200 °C. This could be because the narrow-bandgap SnS_x_ phase in the film was converted to SnO_2_ through annealing [52,53]; the S content in the film was decreased after annealing, as can be seen from the AES results. Due to the roughness of the surface of films, the transmittance results obtained from measurements at different points of the same film have variations of 1–2%, but this error is not so serious to affect the above conclusions.

The films prepared on the alkali-free glass substrate were subjected to resistivity measurement and the calculated resistivity values are listed in Table 2. It was confirmed that the alkali-free glass substrate itself is insulating. Current–voltage (I–V) measurements were performed on three different points for each sample, and the average value of resistivity was calculated. The resistivity before annealing was 1.4 × 10^6^ Ωcm and it decreased to 3.4 × 10^4^ Ωcm after annealing at 200 °C, further decreasing to 1.8 Ωcm after annealing at 400 °C. Thus, as the annealing temperature increases, the film resistivity gradually decreases, which is consistent with the results of SnO_2_ film prepared using other methods [54]. Improvement in crystallinity and/or increase in the amount of oxide vacancies or excess metal ions will lead to a decrease in film resistivity [55].

**Figure 8:**
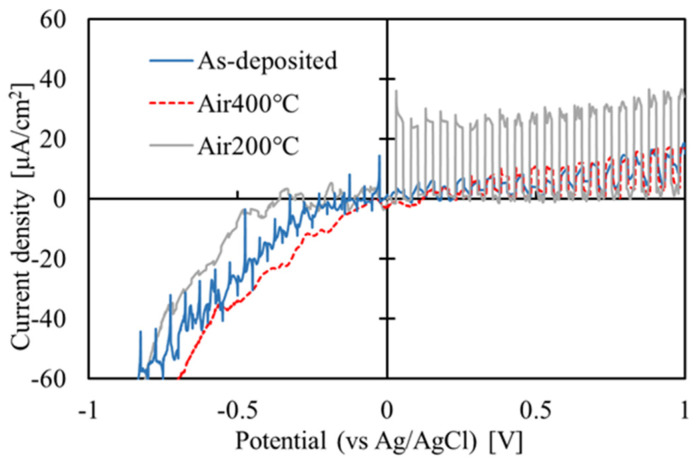
PEC measurement results of the films before and after annealing.

The PEC measurement results of the films before and after annealing are shown in Figure 8. During the positive scan, the properties of the film changed due to the reactions caused by the current, resulting in discontinuity at 0 V between the data of the positive and negative scans. Both before and after annealing, photo response was observed under positive bias, indicating that the films were all n-type. The photo response of the film annealed at 200 °C is significantly larger than those of the films before annealing and those annealed at 400 °C. Therefore, in the following heterojunction experiment, the film was annealed at 200 °C.

SnO_2_ films have so far been prepared using another simple chemical technique, called CBD [22,23]. Following are the differences in deposition results between CBD and DDD. The films prepared using CBD have higher transmittance (about 70–85%), but the resistivity of the films before annealing is very high (not measurable) and the resistivity after annealing at 450 °C is as high as 10^3^ Ωcm. The SnO_2_ films prepared using DDD have conductivity both before and after annealing, and the resistivity of the films after annealing at 400 °C is only 1.8 Ωcm. The transmittance in the visible field is about 50–80%. Thus, the DDD-SnO_2_ films have high conductivity and low transmittance compared with the CBD-SnO_2_ films.

### 3.3. Co_3_O_4_/SnO_2_ Heterojunction

The thickness of the Co_3_O_4_/SnO_2_ heterojunction has been measured to be about 0.15 µm for SnO_2_ and 0.2 µm for Co_3_O_4_. The XRD results of the annealed heterojunction are shown in Figure 6b. In addition to the peaks of the ITO substrate, peaks caused by the films were observed. Comparison with the powder diffraction file ICDD 00-009-0418 shows that these peaks are the (311), (400) and (440) peaks of Co_3_O_4_, respectively. It is consistent with the results obtained from our previous study on Co_3_O_4_ films [30]. Thus, Co_3_O_4_ was successfully deposited onto SnO_2_ to form the Co_3_O_4_/SnO_2_ heterojunction.

**Figure 9:**
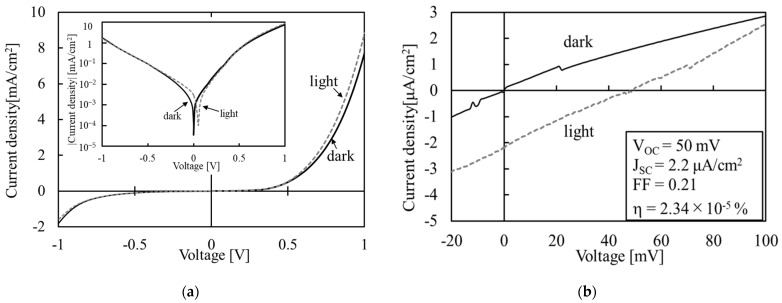
J-V measurement results of the Co_3_O_4_/SnO_2_ heterojunction: (**a**) −1 to 1 V and (**b**) −20 to 100 mV.

Figure 9 shows the J-V characteristics of the Co_3_O_4_/SnO_2_ heterojunction in dark and under AM1.5 (100 mW/cm^2^). In Figure 9a, rectification characteristics and photo-responsivity are observed. As shown in the enlarged figure (Figure 9b), its open-circuit voltage V_oc_ is about 50 mV and its short-circuit current J_sc_ is about 2.2 μA/cm^2^. The calculated energy conversion efficiency is about 2.3 × 10^−5^%. The above results indicate that Co_3_O_4_/SnO_2_ heterojunctions can be used as diodes and solar cells. However, its leakage current is seriously large, which affects its performance in solar cells.

As noted in Introduction, there are only a few reports on SnO_2_-based p-n heterojunction diodes; SnO_2_ has been mainly used as transparent electrodes, dye-adsorbed films of dye-sensitized solar cells or electron selective layers of perovskite solar cells [15,16,17,18,19,20,21]. SnO_2_-based heterojunctions have been mostly used in catalysts and sensors [41,42,43,44,45,46,47]. In this study, it was shown for the first time that a Co_3_O_4_/SnO_2_ heterojunction is applicable in a solar cell. And due to the wide bandgap and transparency, SnO_2_ can be combined with a wide bandgap p-type semiconductor, such as NiO, to fabricate transparent solar cells. In addition, SnO_2_ prepared using DDD can also be used to prepare low-cost transparent electronic devices, such as transparent diodes [56] and transparent thin-film transistors (TFT) [57]. Those devices can be prepared on a non-conductive substrate, such as glass at low temperatures using DDD, which will drastically reduce the fabrication cost.

Although Co_3_O_4_/SnO_2_ heterojunction solar cells prepared via DDD are not efficient at present, they can be applied in the field of internet of things (IoT) devices. It was reported that perovskite solar cells can power wireless devices (radio frequency identification), which required 10–45 µW of power [58]. Gas sensors based on the WS_2*x*_Se_2–2*x*_ alloy requires only 0.75–21 μW of power to operate [59]. A self-powered visible-light-transparent CO_2_ gas sensor based on a NiO/ZnO solar cell was also reported [60]. Such low-power IoT devices are increasingly used in daily life, and solar cells are useful as a power source even if they are not of high-efficiency.

For future research, the quality of the SnO_2_ films should be improved for better performance of solar cells, and their applications need to be examined more, such as in transparent diodes, transistor, and solar cells.

## 4. Conclusions

In this study, tin hydroxide films were successfully prepared via DDD using Na_2_S_2_O_3_ as the complexing agent and were then converted into SnO_2_ films through annealing. The complexing agent was used for the first time to suppress spontaneous hydrolysis and precipitation in the deposition solution.

The transmittance of the annealed films was higher than 50% in the visual range. The results of the AES measurement showed that the O/Sn composition ratio of the annealed films approached two, indicating that the annealed films were SnO_2_. The PEC results showed that the annealed films were n-type, and the photo response of the films annealed at 200 °C was significantly larger than that of the films annealed at 400 °C. And the results of resistivity measurement showed that the films annealed at 400 °C have a lower resistivity of 1.8 Ωcm.

In this study, Co_3_O_4_/SnO_2_ p-n heterojunctions were fabricated with Co_3_O_4_ deposited via DDD, and rectification and photovoltaic properties were observed. It was proven that Co_3_O_4_/SnO_2_ heterojunctions can potentially be used in solar cells. Although the energy conversion efficiency was still low, about 10^−5^%, the cell could be used in low-power IoT applications. Moreover, it is demonstrated for the first time that oxide thin film solar cells can be fabricated using only DDD. The metal oxide materials, such as SnO_2,_ are abundant, and the equipment required for DDD is very simple and low cost. Thus, this work could contribute to reducing the fabrication cost of various thin film devices, especially in the field of transparent electronics.

## Figures and Tables

**Table 1:** Solution conditions with Na_2_S_2_O_3_.

Condition	SnSO_4_ [mM]	Na_2_S_2_O_3_ [mM]	NaOH [mM]	pH	Precipitate	Solution Color	Thickness [μm]
I	10	50	—	3.8	none	yellowish-white	0.2
II	10	100	—	3.5	none	white	0.2
III	10	200	—	3.4	none	Colorless	—
IV	20	50	—	4.4	yellow	yellow	—
V	10	50	2.5	4.7	yellow	yellow	—
VI	10	50	25	11.7	white→black	white→dark	—

**Table 2:** The O/Sn composition ratio and resistivity of the thin film before and after annealing.

	As-Deposited	200 °C	400 °C
O/Sn	1.11	1.65	2.16
resistivity (Ωcm)	1.4 × 10^6^	3.4 × 10^4^	1.8

## Data Availability

The data are contained within the article.
